# Robotically Assisted vs. Manual Total Hip Arthroplasty in Developmental Hip Dysplasia: A Comparative Analysis of Radiological and Functional Outcomes

**DOI:** 10.3390/jcm14020509

**Published:** 2025-01-15

**Authors:** Hakan Zora, Gökhan Bayrak, Ömer Faruk Bilgen

**Affiliations:** 1 Department of Orthopedics and Traumatology, Private Medicabil Hospital, 16140 Nilüfer/Bursa, Türkiye; mdhakanzora@gmail.com (H.Z.); ofbilgen@gmail.com (Ö.F.B.); 2 Department of the Physiotherapy and Rehabilitation, Faculty of Health Sciences, Muş Alparslan University, 49250 Muş Merkez/Muş, Türkiye

**Keywords:** developmental dysplasia of the hip, coxarthrosis, total hip arthroplasty, robotic surgical procedures, surgical outcomes

## Abstract

**Background/Objectives**: Developmental dysplasia of the hip (DDH), defined by the malalignment of the femoral head and acetabulum, is a major precursor to coxarthrosis, posing substantial challenges during total hip arthroplasty (THA). Patients with coxarthrosis secondary to DDH often exhibit acetabular bone insufficiency, which makes challenging surgical reconstruction difficult. This study aimed to compare the radiologic and functional outcomes of robotically assisted and conventional manual THA techniques in patients with coxarthrosis secondary to Crowe type III–IV DDH. **Methods**: This prospective study included 40 patients divided into robotically assisted (n = 20) and conventional manual (n = 20) THA groups. Evaluations encompassed hip pain (Visual Analogue Scale, VAS), function (Harris hip score and University of California, Los Angeles, activity scale), quality of life (Short Form-12), and prosthesis sensation (Forgotten Joint Score-12). Radiologic outcomes included acetabular inclination and anteversion angles. Femoral shortening, operative duration, and follow-up times were also analyzed. **Results**: Demographic characteristics did not differ between groups (*p* > 0.05). Robotically assisted THA exhibited a significantly longer operative time (171.40 ± 11.96 vs. 150.30 ± 14.67 min; *p* = 0.001) but a shorter follow-up (29.3 ± 8.51 vs. 52.95 ± 18.96 months; *p* = 0.001), without a difference in the amount of femoral shortening (*p* = 0.947). Despite the extended surgical duration, the two techniques achieved comparable radiologic outcomes, with no significant differences in acetabular inclination or anteversion angles (*p* > 0.05). Functional assessments, including Harris hip scores (73.85 vs. 73.95; *p* = 0.978), UCLA activity scores, and VAS, indicated similar efficacy between groups. SF-12 physical and mental quality of life and Forgotten Joint Score-12 prosthesis sensation did not differ between groups (*p* > 0.05). **Conclusions**: This study concludes that robotically assisted and conventional manual THA present similar radiologic and functional outcomes in patients with coxarthrosis secondary to Crowe type III–IV DDH, as displayed by comparable acetabular anteversion and inclination alignment, femoral shortening, hip function, pain, quality of life, and prosthesis sensation scores. While robotically assisted THA requires a longer operative time, its precision in implant placement may hold potential advantages for long-term outcomes, demanding further investigation in extended follow-up studies.

## 1. Introduction

Developmental dysplasia of the hip (DDH) is defined as the presence of the femoral head in a different location from its normal relationship with the acetabulum [1]. While total hip arthroplasty (THA) is a proven procedure for alleviating pain and enhancing hip function in coxarthrosis, the implementation of THA after coxarthrosis secondary to DDH is a topic of significant importance, given the difficulties it presents [2,3]. Patients with coxarthrosis secondary to DDH exhibit acetabular bone insufficiency, which is often encountered during THA, and this may cause significant difficulties in reconstruction [3]. Identification and preparation of the true acetabulum, femoral canal preparation, and the components’ reduction present significant technical problems during THA surgery [4]. Despite all the difficulties in its implementation, many surgeons often prefer THA for coxarthrosis secondary to DDH [5].

Conventional manual THA places acetabular components according to surgeon experience and mechanical alignment guidelines. However, over the last twenty years, the accuracy of these methods has been questioned [6]. A literature review showed that free and mechanically guided techniques, even when used by experienced surgeons, can result in tilt, anteversion angles, and incorrect cup positions with the acetabular component placed outside the defined safe zone [6,7]. Recent innovations have brought about new approaches for THA surgery, and robotically assisted surgery has been utilized over the last two decades [8]. It aims to improve functional results and implant survival by enabling more precise component positioning based on the patient’s individual three-dimensional anatomy [9,10]. Robotically assisted surgery enables more precise and consistent control over acetabular component positioning during THA [11]. The system uses preoperative CT scans to establish a reference plane based on the patient’s supine functional position, which has been shown to improve the likelihood of achieving cup placement within the traditional safe zone [12].

Several investigations have reported better functional results in robotically assisted arthroplasty procedures [10,11,13] achieved by higher accuracy, translating into a lower implant failure rate [14]. Hayashi et al. [15] investigated the accuracy of implant placement in robotically assisted THA patients. The authors indicated that severe DDH cases, including Crowe type III–IV, were rare and concluded that they needed to be analyzed. Additionally, compared to conventional surgery, robotically assisted surgery’s effect on patient-reported outcomes has not been elicited [16]. Considering the deteriorated and complex bone and soft-tissue status after coxarthrosis secondary to Crowe type III–IV DDH, the effects on radiological and functional outcomes in robotically assisted and manual THA interventions, which may offer advantages in implant placement, need to be explored. This study aimed to compare radiologic and functional outcomes following robotically assisted and conventional manual THA in patients with coxarthrosis secondary to Crowe type III–IV DDH.

## 2. Methods

### 2.1. Study Design

This prospective study was conducted in the orthopedics and traumatology clinic of the Private Medicabil Hospital. The İstanbul Yeni Yüzyıl University Health Sciences Research Ethics Committee confirmed the ethical approval of the study (2024/11-1352). The study was carried out under the principles of the Declaration of Helsinki. Informed consent was obtained from all patients.

### 2.2. Patients

We identified patients who underwent THA due to coxarthrosis secondary to Crowe type III–IV DDH at the Private Medicabil Hospital between 2016 and 2024 from the hospital’s surgical operation database. From 2016 to 2020, only conventional manual THA was performed at our hospital. In 2020, we introduced MAKO^®^ robotic arm-assisted total hip arthroplasty (Stryker, Kalamazoo, MI, USA). After this introduction, we provided detailed explanations of both surgical techniques to our patients, allowing them to choose their preferred method for the procedure. Patients who underwent THA using robotically assisted or conventional manual procedures were recorded. The inclusion criteria were admitted patients undergoing THA due to coxarthrosis secondary to Crowe III-IV DDH and those undergoing femoral shortening aged 20–75 who did not have any psychiatric disorder that may have affected their responses. The exclusion criteria were revision due to periprosthetic or vascular complications, superficial and deep infections, dislocations, and sciatic nerve damage.

The power ratio of the sample to be selected was calculated based on a previous study [3], and the effect size was determined as d = 0.48. According to the power analysis, assuming that we could achieve a similar effect size (d = 0.48), an α level of 0.05, and a power of 0.80, at least 18 patients in the robotically assisted THA group and at least 18 patients in the conventional manual THA group were required to complete the study. From 2016 to 2024, 30 conventional manual and 32 robotically assisted THA procedures secondary to Crowe type III–IV DDH were performed at our hospital. In the conventional manual THA group, one patient was excluded due to revision surgery after early postoperative dislocation; one patient had a superficial infection and wound debridement; five patients were excluded because they lived in another country; and one patient suffered sciatic nerve damage, which healed by the eighth postoperative week. In the robotically assisted THA group, the first five cases were excluded due to the potential learning curve effect; one patient was excluded due to close reduction after early postoperative dislocation; one patient had a deep infection; two patients were excluded because they lived in another country; and one patient suffered sciatic nerve damage, which healed by the tenth postoperative week. A proportion of 25% more than the targeted number of participants (22 patients for each group) were called and invited for measurements and evaluations in the case of dropout. However, two patients in each group did not want to participate in the assessments. Ultimately, 20 patients in the robotically assisted THA group and 20 patients in the conventional manual THA group were included in the study.

### 2.3. Surgical Techniques

**Figure 1:**
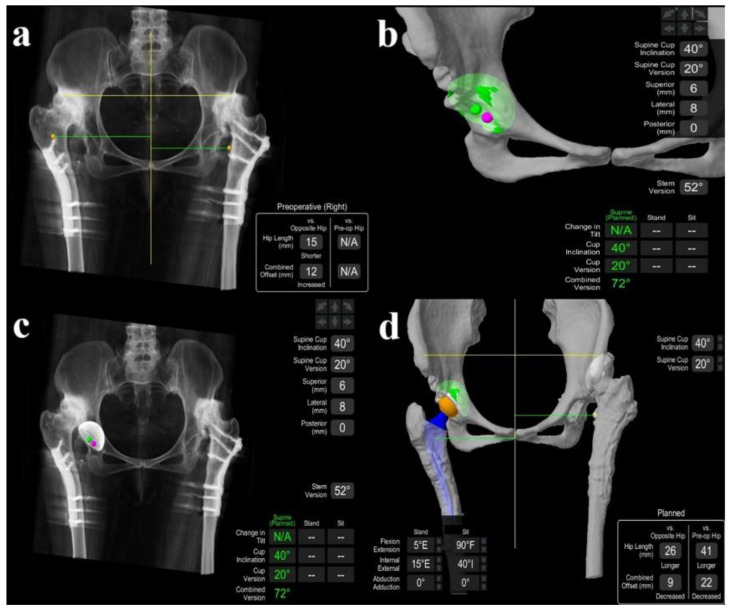
(**a**) Preoperative length and offset planning; (**b**) angular planning of preoperative acetabular cup anteversion and inclination; (**c**) preoperative acetabular cup planning; (**d**) preoperative combined acetabular and femoral component planning.

All the surgical interventions were conducted with one experienced surgeon and the same surgical team. The surgeon has more than 30 years of surgical experience and performs more than 50 robotically assisted THAs and more than 50 conventional THAs annually. For both surgical techniques, preoperative CT images of the pelvis, hips, and knees were prepared. In the MAKO^®^ robotic arm-assisted system, detailed preoperative planning was performed by uploading CT images to the virtual environment to select the appropriate size component and place the acetabular and femoral components at the correct angles (Figure 1).

#### 2.3.1. Robotically Assisted Surgical Technique

**Figure 2:**
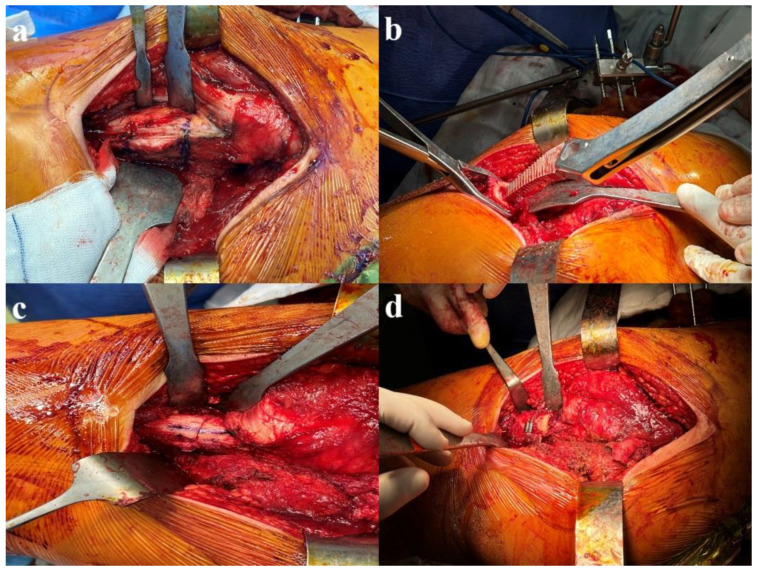
(**a**) Determination of the femoral amount of shortening; (**b**) distal femoral canal preparation; (**c**) control of femoral rotation; (**d**) placement and fixation of the bone tissue on the osteotomy line.

**Figure 3:**
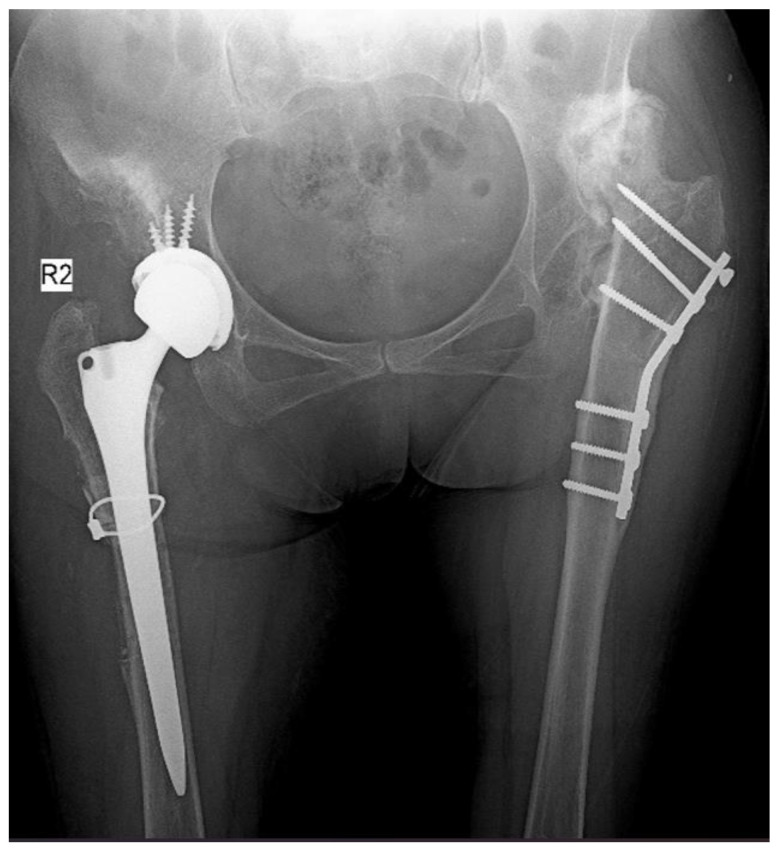
Postoperative X-ray image of the patient whose preoperative planning was performed using a robotically assisted surgical technique.

The patient was placed on the operating table in the lateral decubitus position with the ipsilateral iliac wing and knee joint open. First, three Schanz screws were sent anteriorly from the thickest part of the iliac wing. The robotic sensor was then placed. A posterior approach was utilized to open the hip joint. Checkpoint sensors were placed on the greater trochanter and acetabulum. The surgeon defined the required points using a special probe. The femoral head was resected using the robotic incision arm. The actual acetabulum was identified and prepared. Rasping of the acetabulum was performed with the assistance of the robotic arm. The femoral head was used as a graft in patients with bone defects. The appropriate acetabular component was placed using robotic arm support. After stability control, fixation was performed with three screws. After the placement of the polyethylene, soft tissue loosening was completed. Then, the femoral canal was prepared. Reduction was attempted with a trial prosthesis, and the required amount of shortening was determined. The vastus lateralis was bluntly displaced anteriorly distal to the trochanter major for femoral shortening. The amount of femoral shortening (Figure 2a) and the rotation control line were measured and marked with a ruler. The osteotomized bone tissue was displaced anteriorly and posteriorly in two pieces without separating the soft tissues. The femoral canal preparation was renewed (Figure 2b), and the planned femoral component was placed. Hip reduction was performed, and femoral rotation was controlled (Figure 2c). Component angles, limb length, and offset were checked using the MAKO^®^ robotically assisted system. Bone tissues were placed in a cylinder shape on the osteotomy line and fixed with a cable (Figure 2d). The posterior structures were then repaired, and the incision was closed. Figure 3 shows the postoperative X-ray image of the patient whose preoperative planning was performed, as described above, using a robotically assisted surgical technique.

#### 2.3.2. Conventional Manual Surgical Technique

The patient was placed on the operating table in the lateral decubitus position with the ipsilateral iliac wing and knee joint open. A posterior approach was utilized to open the hip joint using a surgical saw. The actual acetabulum was identified and prepared after the femoral head was excised. The femoral head was used as a graft in patients with bone defects. The appropriate acetabular component was fixed using screws. After the placement of the polyethylene, soft tissue loosening was completed. Then, the femoral canal was prepared. Reduction was attempted with a test prosthesis, and the required amount of femoral shortening was determined. The vastus lateralis was bluntly displaced anteriorly distal to the trochanter major for femoral shortening. The amount of shortening and rotation control lines were measured and marked with a ruler. The osteotomized bone tissue was displaced anteriorly and posteriorly in two pieces without separating the soft tissues. The femoral canal preparation was renewed, and the planned femoral component was placed. Hip reduction was performed, and femoral rotation was controlled. Bone tissues were placed in a cylinder shape on the osteotomy line and fixed with cable. Then, the posterior structures were repaired, and the incision was closed.

### 2.4. Outcome Measures

Patient demographic data, such as age, height, weight, gender, operated extremity, and Crowe type, were recorded on the data form. The Visual Analogue Scale (VAS, 0–10) was used to evaluate the hip pain status of the patients. University of California, Los Angeles (UCLA) activity scale score [17] and Harris hip score [18] were used to evaluate hip function. The Short Form-12 (SF-12) was used to assess quality of life [19], and the Forgotten Joint Score-12 was used to determine the normal sensation of the prosthesis [20]. Using Widmer’s method [21], acetabular inclination and anteversion angles were recorded for radiological comparisons. Shortening amounts (mm) were calculated intraoperatively using a ruler. The mean follow-up and surgical duration were also documented.

### 2.5. Statistical Analysis 

Data obtained in the study were analyzed statistically using IBM SPSS 27.0 software (IBM SPSS Statistics for Windows, Armonk, NY, USA). The conformity of the data to a normal distribution was examined with the Shapiro–Wilk test. Descriptive statistics are reported as mean ± standard deviation values for continuous variables and as number (n) and percentage (%) for categorical variables. An independent-samples *t*-test was used to explore the means between the two groups. The Pearson chi-square test was used for categorical data analyses. The statistical significance level was set as *p* < 0.05.

## 3. Results

A total of 40 patients’ data were analyzed in the study. The mean follow-up was 29.3 ± 8.51 months in the robotically assisted and 52.95 ± 18.96 months in the conventional manual THA group (*p* = 0.001). The mean surgical duration was 171.40 ± 11.96 min in the robotically assisted and 150.30 ± 14.67 min in the conventional manual THA group (*p* = 0.001). Nineteen patients (95%) had neutral femoral stem alignment, and only one patient (5%) in both groups had varus femoral stem alignment (*p* = 1.000).

Table 1 presents a comparison of demographic characteristics between the two groups. The robotically assisted group comprised of 2 males (10%) and 18 females (90%), and the conventional manual group included 2 males (10%) and 18 females (90%) (*p* = 1.000). In terms of Crowe types, 11 were type III (55%) and 9 were type IV (45%) in the robotically assisted group, while 12 were type III (60%) and 8 were type IV (40%) in the conventional manual group (*p* = 0.749). With respect to operated extremities, 14 were dominant (70%) and six were non-dominant (30%) extremities in the robotically assisted group, and 13 were dominant (65%) and seven were non-dominant (35%) in the conventional manual group (*p* = 0.736). The mean age was 63.75 years in the robotically assisted group and 61.50 years in the conventional manual THA group (*p* = 0.729). Similarly, the body mass index did not differ, at 22.57 kg/m^2^ in the robotically assisted group and 24.59 kg/m^2^ in the conventional manual THA group (*p* = 0.850). Importantly, there were no significant differences in the number of chronic diseases, with 1.25 in the robotically assisted group and 1.5 in the conventional manual group (*p* = 0.537).

Table 2 presents a comparison of functional clinical data between the two groups. Notably, the acetabular inclination and anteversion angles demonstrated no significant differences, with *p*-values of 0.519 and 0.640, respectively. This consistency is further reflected by the similar shortening of the femur across groups, achieving a *p*-value of 0.947.

When examining pain levels during activity, the VAS scores support the effectiveness of both approaches, revealing scores of 13.6 mms in the robotically assisted group and 14.2 mms in the conventional manual THA group (*p* = 0.635). The UCLA activity scores were also comparable, at 22.7 for the robotically assisted group and 22.4 for the conventional group (*p* = 0.778). The Harris hip scores were almost identical, with the robotically assisted group at 73.85 and the conventional group at 73.95 (*p* = 0.978). The Forgotten Joint Score-12 reflected a similar outcome, showing values of 39.79 and 39.89, respectively, with a *p*-value of 0.980 (Table 2).

Finally, the SF-12 physical and mental quality-of-life scores further confirm the efficacy of robotically assisted and conventional manual techniques, with physical scores at 40.29 and 40.32 (*p* = 0.987) and mental scores at 54.01 and 55.88 (*p* = 0.233). These data strongly suggest that robotically assisted and conventional approaches yield comparable clinical outcomes, reinforcing their value in clinical practice (Table 2).

## 4. Discussion

The purpose of this comprehensive study was to compare radiologic and functional outcomes following robotically assisted or conventional manual THA in patients with coxarthrosis secondary to Crowe type III–IV DDH. Our observations of radiologic outcomes, including acetabular inclination and anteversion angles and the amount of shortening, and functional outcomes regarding the Harris hip score, UCLA activity score, pain, quality of life, and normal feeling of the prosthesis led to the conclusion that the two groups exhibit equivalent results. However, the mean operative time was longer for robotically assisted THA than for conventional manual THA.

Radiologic alignment is an essential issue for THA surgery regarding prosthesis survival. Lewinneck et al. [22] proposed that the “safe zone” of the acetabular component should have a radiographic inclination of 40° ± 10° and an anteversion of 15° ± 10°. Similarly, Sugano et al. [23] reported an optimal acetabular component target angle of 36–45° radiographic inclination and 10–24° anteversion to prevent dislocation and diverse complications after THA. An earlier investigation comparing robotically assisted and conventional manual THA for DDH patients with Crowe classifications I–IV reported no difference between groups in cup anteversion or inclination angles [12]. The present study supports the previous findings, as the inclination and anteversion angles were 40.41° and 19.17°, respectively, in the robotically assisted group and 39.96° and 18.74°, respectively, in the conventional THA group, indicating the safe zone for implant alignment. Another important finding is that severe dysplasia, including type Crowe III and IV, mainly requires femoral shortening to safely reposition the hip to its anatomic position [24]. Our results suggest that robotically assisted (2.77 cm) and conventional manual (2.76 cm) THA had almost equal femoral shortening distances for THA implementation during the surgery. Surgeon expertise, prosthesis customization, and standardized surgical goals may have led to similar radiologic alignments for robotically assisted and conventional manual surgical techniques for THA surgery.

Performing THA on DDH patients requires specific effort due to the challenges and complexity inherent to the surgical procedures; therefore, the operative time for DDH patients could be longer [8,25]. Prior investigations reported mean operation times of 94.4 [26] and 111.5 [27] minutes in DDH patients using the conventional manual THA technique. Introducing new techniques and technology to the surgery, including robotically assisted THA, can cause increased operative times [8]. Domb et al. [28] reported 162.3 min for robotically assisted THA and 158.8 min for conventional manual THA in osteoarthritic patients. Zhou et al. [12] reported that the operative time for robotically assisted THA was 85.3 min, while that for conventional manual THA was 88.6 min for DDH patients with Crowe classifications I–IV. Our results indicate that the mean surgical duration was 171.4 min in the robotically assisted group and 150.3 min in the conventional manual THA group. Since femoral shortening was performed in all patients in our study, the mean surgical duration of the groups was higher than previous findings. Surgical workflow integration, patient-specific complexity, and prosthesis customization may have led to extended surgical time in the robotically assisted group. In addition, the increased mean surgical duration in the robotically assisted group may have hindered its potential to achieve better outcomes regarding pain and function.

Prior studies have noted the importance of functional improvement after THA in DDH patients [25,29,30], mainly occurring in the first year after surgery and resolving in the following years [29]. Several studies investigating functional Harris hip scores after conventional THA implementation for DDH patients, indicating fair [30] and good results [31,32]. Bukowski et al. [13] investigated robotically assisted vs. conventional THA and found excellent vs. good results, respectively, on the Harris hip score [33]. Likewise, the UCLA activity scores of DDH patients who underwent THA indicated low [15] to high activity levels [34] according to Murrey et al. [35]. Our study found that the Harris hip scores for the robotically assisted and conventional manual THA groups were fair, aligning closely with findings in the existing literature. The unidentified potential baseline similarity of the functional scores, the experienced surgeon’s high level of expertise in the conventional manual technique, the potentially prolonged learning curve effect, the role of prosthesis customization, and the limitations of the assessment tools may have led to the achievement of similar functional outcomes between the two surgical techniques. However, the UCLA activity scores of our sample indicated high activity levels for both the robotically assisted and conventional THA groups, indicating superior outcomes compared to the earlier findings.

There is a growing consensus that THA in patients with DDH yields substantial improvements in pain relief and quality of life [24]. Hip pain in DDH patients significantly decreased following conventional THA, with a VAS score of 1.03 cm after one year [36] and 3.4 cm after two years [26]. In comparison, robotically assisted THA yielded a VAS score of 0.7 cm two years post-surgery [37]. The current study similarly found near-equivalent VAS pain scores, with 1.36 cm in the robotically assisted group and 1.42 cm in the conventional THA group, suggesting a lower pain level than other findings. Prior research also reports that SF-12 physical and mental quality-of-life scores showed no significant difference between robotically assisted and conventional THA procedures at long-term follow-up [13]. A systematic review and meta-analysis also reported no significant differences in pain and quality of life between robotically assisted and conventional manual THA [16]. Quality-of-life outcomes for the two groups in this study also showed similar results. The unknown potential baseline similarity of the SF-12 scores, the high level of expertise of the experienced surgeon, and the complexity of DDH pathophysiology may have prevented the possible advantage of robotically assisted surgery in improving patients’ quality of life.

Unlike other patient-reported outcome scales, the Forgotten Joint Score-12 emphasizes patients’ awareness of their newly created artificial joints. This outcome highlights implant integration into patients’ lives, providing deeper insights into their overall satisfaction post-surgery [20]. Masson et al. [38] analyzed functional outcomes after conventional THA in DDH patients, and they found that the forgotten joint score was 79.6 points and 86.2% of patients were satisfied after twelve years of follow-up. Prior robotically assisted THA investigations yielded a forgotten joint score of 83.1 at two-year follow-up [37]. An earlier systematic analysis discovered no significant difference between robotically assisted and conventional THA surgeries on forgotten joint scores [11]. For the first time, comparing robotically assisted and conventional THA surgeries on forgotten joint scores in DDH patients, this study showed almost identical results. These findings challenge the notion that one technique may significantly outperform the other, highlighting the effectiveness of both methods in restoring a normal feeling of the new artificial joint for patients.

This study had several limitations. First, the patients’ physical activity levels were not questioned. Additionally, the differences in the follow-up periods between the study groups and the small size of the study population are significant limitations. Lastly, we lacked data on the patients’ preoperative, early postoperative, and long-term functional outcomes.

## 5. Conclusions

This study demonstrates that robotically assisted and conventional manual THA yield comparable functional and radiologic outcomes in patients with coxarthrosis secondary to Crowe type III–IV DDH. Despite the longer operative time required for robotically assisted surgery and its ability to achieve precise anatomic “safe zone” alignment without providing significant functional benefits in the early-to-midterm follow-up, this study emphasizes the requirement to consider the robotically assisted technique’s role in clinical practice. While the precise anatomical alignment achieved with robotically assisted surgery may hold promise for improving long-term implant survival, further research involving a larger patient population and extended follow-up periods and considering physical activity levels and preoperative functional assessments is required.

## Figures and Tables

**Table 1:** Demographic data of the groups.

Variable	Robotically Assisted THA (n = 20)		Conventional Manual THA (n = 20)		
	Mean ± SD	95% CI	Mean ± SD	95% CI	*P* ^1^
Age (years)	63.75 ± 6.13	53.99–73.50	61.50 ± 11.90	42.56–80.43	0.729
Body mass index (kg/m^2^)	22.57 ± 1.67	19.91–25.24	24.59 ± 1.26	22.58–26.61	0.435
Number of chronic diseases	1.25 ± 0.5	0.45–2.04	1.50 ± 0.57	0.58–2.41	0.537
	**n**	**%**	**n**	**%**	***P* ^2^**
Gender					
Female	18	90	18	90	1.000
Male	2	10	2	10	
Crowe Types					
Type III	11	55	12	60	0.749
Type IV	9	45	8	40	
Operated Extremity					
Dominant	14	70	13	65	0.736
Non-dominant	6	30	7	35	

SD: standard deviation; 95% CI: 95% confidence interval for means; kg: kilogram; m: meter; *P*^1^: independent-samples *t*-test significance value; *P*^2^: Pearson chi-square test statistical significance value.

**Table 2:** Functional clinical data comparison between the groups.

Variable	Robotically Assisted THA (n = 20)		Conventional Manual THA (n = 20)		
	Mean ± SD	95% CI	Mean ± SD	95% CI	*P*
Acetabular inclination angle	40.41 ± 2.67	39.15–41.66	39.96 ± 1.47	39.27–40.65	0.519
Acetabular anteversion angle	19.17 ± 2.87	17.83–20.52	18.74 ± 2.98	17.35–20.14	0.640
Amount of femoral shortening (cm)	2.77 ± 0.45	2.56–2.98	2.76 ± 0.48	2.53–2.99	0.947
VAS activity pain (cm)	1.36 ± 0.41	1.16–1.55	1.42 ± 0.37	1.24–1.59	0.635
UCLA score	22.70 ± 3.45	21.08–24.31	22.40 ± 3.21	20.89–23.90	0.778
Harris hip score	73.85 ± 11.87	68.29–79.40	73.95 ± 10.60	68.98–78.91	0.978
Forgotten Joint Score-12	39.79 ± 12.91	33.75–45.84	39.89 ± 12.59	34.01–45.79	0.980
SF-12 physical score	40.29 ± 3.87	38.47–42.11	40.32 ± 5.04	37.96–42.68	0.987
SF-12 mental score	54.01 ± 5.20	51.58–56.45	55.88 ± 4.49	53.77–57.98	0.233

SD: standard deviation; 95% CI: 95% confidence interval for means; kg: kilogram; m: meter; *P*: independent-samples *t*-test significance value.

## Data Availability

Due to privacy and ethical restrictions, the data are not publicly available.
